# α-1,6-Fucosyltransferase Is Essential for Myogenesis in Zebrafish

**DOI:** 10.3390/cells12010144

**Published:** 2022-12-29

**Authors:** Nozomi Hayashiji, Genri Kawahara, Xing Xu, Tomohiko Fukuda, Aurelien Kerever, Jianguo Gu, Yukiko K. Hayashi, Eri Arikawa-Hirasawa

**Affiliations:** 1 Center for Genomic and Regenerative Medicine, Juntendo University Graduate School of Medicine, Tokyo 113-8421, Japan; 2 Department of Pathophysiology, Tokyo Medical University, Tokyo 160-8402, Japan; 3 Division of Regulatory Glycobiology, Institute of Molecular Biomembrane and Glycobiology, Tohoku Medical and Pharmaceutical University, Miyagi 981-8558, Japan; 4 Research Institute for Diseases of Old Age, Juntendo University Graduate School of Medicine, Tokyo 113-8421, Japan

**Keywords:** α-1,6-fucosyltransferase, zebrafish, muscle development

## Abstract

Glycosylation is an important mechanism regulating various biological processes, including intercellular signaling and adhesion. α-1,6-fucosyltransferase (Fut8) belongs to a family of enzymes that determine the terminal structure of glycans. Fut8 is widely conserved from Caenorhabditis elegans to humans, and its mutants have been reported in humans, mice, and zebrafish. Although mutants show various symptoms, such as spinal deformity and growth retardation, its effects on skeletal muscles are unknown. We aimed to elucidate the function of Fut8 in skeletal muscle using zebrafish and C2C12 cells for evaluation. We observed that most fut8a morphants died at 2 days post-fertilization (dpf) or in earlier developmental stages even at low concentrations of morpholino oligonucleotides (MOs). Mutant juveniles also had small body sizes, and abnormal myocepta and sarcomere structures, suggesting that Fut8a plays important roles in myogenesis. Moreover, treatment of C2C12 cells with 2-fluorofucose (2FF), a fucosylation inhibitor, during cell differentiation dramatically reduced the expression of myogenic genes, such as *Myomaker* and other myogenic fusion genes, and inhibited myotube formation. These results indicate that Fut8 is an important factor in myogenesis, and myofusion in particular.

## 1. Introduction

Glycosylation is a commonly occurring post-translational modification of proteins, and many eukaryotic proteins exist as glycoproteins that are modified by glycans [1,2]. The same glycoprotein can be modified by different glycans in different tissues and cells, making glycosylation regulation an extremely complex process. The addition of glycans to proteins enables them to perform various functions, such as intercellular signal transduction and regulation of the degradation rate of proteins [3,4].

Fucosyltransferases are enzymes that regulate fucosylation, a type of glycosylation. They determine the glycan end structure rather than the branching structure of the glycan and are classified into α-1,2-fucosyltransferases, α-1,3-fucosyltransferases, and α-1,6-fucosyltransferase (Fut8), based on differences in substrate characteristics and reaction specificities [5]. Many studies have been conducted on α-1,2- and α-1,3-fucose transfer groups, and have reported their involvement in the synthesis of glycan epitopes essential for blood group antigens and selection-based recognition between immune cells [6]. Fut8 is responsible for catalyzing core fucosylation, which in turn is responsible for the transfer of fucose to N-acetylglucosamine (GlcNAc) in the α-1,6 linkage at the innermost GlcNAc residue of N-glycans [7]. α-1,2- and α-1,3-fucosyltransferases are interchangeable, while Fut8 is the only enzyme for core fucosylation. Fut8 is widely conserved, from Caenorhabditis elegans to humans, suggesting that core fucose is important for protein function. Many pivotal glycoproteins in mammalian tissues are core fucosylated, and core fucosylation is essential to the functions of these proteins: E-cadherin, integrin, epidermal growth factor (EGF) receptor, transforming growth factor (TGF)-β receptor, c-mesenchymal-epithelial transition factor (c-Met), and Fms-like tyrosine kinase 3 (FLT3) [8,9,10,11,12]. Dysfunction of Fut8 has been reported in humans, mice, and zebrafish. In humans, these mutants exhibit marked growth retardation, respiratory failure, neurological symptoms, and dysphagia [13,14]. In mice, most mutants die within three days after birth, and surviving mice have growth defects, emphysema-like alveolar abnormalities [10], and neuronal disorders [15]. Zebrafish injected with morpholino oligonucleotides (MOs) for fut8a develop eye dysplasia and abnormal midline patterning during development [16]. None of these studies described the skeletal muscle in these mutants, however, Fut8 may be involved in myogenesis, as immunohistochemical staining of zebrafish morphants with a skeletal muscle myosin-specific antibody, F59, showed distorted myofibers. Moreover, the symptoms of Fut8 mutant humans resemble those of patients with muscular dystrophy. Zebrafish have some good models of muscle diseases [17,18], which are used for therapeutic drug screens [19]. Gene-specific knockdown can easily be achieved in zebrafish using MO, and their development can easily be observed under a microscope because their fertilized eggs are transparent, making them an excellent model system for investigating the function of target genes during development. Therefore, we used zebrafish to analyze the function of Fut8a in myogenesis.

## 2. Materials and Methods

### 2.1. Morpholino Oligonucleotide Injections

Anti-sense MOs were targeted to disrupt the expression of *fut8a* mRNA. The sequence for *fut8a* MO was 5′-CGCCTACTGCTGCCCTCCCCTTTC-3′, and that for the control morpholino (CMO) was 5′-CCTCTTACCTCAGTTACAATTTATA-3′ (GeneTools, LLC. Philomath, OR, USA). Similar quantities of MOs for *fut8a* and control were injected into the yolk of one- to two-cell-stage embryos. The above sequences were obtained from a previous report [16]. 

### 2.2. Fish Culture

Zebrafish were cultured at 28.5 °C according to standard procedures and criteria [20]. Fertilized eggs were collected and injected with MO, and tricaine solution was used for anesthesia and euthanasia. This research was approved by the Institutional Animal Care and Use Committee (IACUC) of the Tokyo Medical University animal facility (approval number: R3-0029).

### 2.3. Birefringence Assay

The birefringence assay detects skeletal muscle structure abnormalities; an anesthetized fish is placed on a polarizing filter and then covered with a second polarizing filter. In this work, the filter was placed on a dissecting microscope (Leica. Wetzlar, Germany) under light, and the top polarizing filter was adjusted until only the light refracted through the muscle was visible. Because the degree of birefringence is affected by the horizontal orientation of the fish, the fish was rocked back and forth to adjust its position.

### 2.4. Immunohistochemistry

Whole fish embryos were fixed in 4% paraformaldehyde (PFA) overnight at 4 °C and stored in 100% methanol at −20 °C. After rehydration with 50% methanol and blocking with 1% bovine serum albumin (FUJIFILM Wako Pure Chemical Co. Osaka, Japan) in TBS containing 0.05% Tween 20 (TBS-T) (Sigma-Aldrich Co. St. Louis, MI, USA) to reduce non-specific binding, embryos were incubated separately with anti-beta dystroglycan (1:100, Novocastra. Wetzlar, Germany), anti-laminin (1:50, Sigma-Aldrich Co. St. Louis, USA), and anti-myosin heavy chain antibodies (F59, 1:25, Santa Cruz Biotechnology. Dallas, TX, USA) at 4 °C overnight. After several washes with TBS-T, the samples were incubated with secondary antibodies (1:500, anti-mouse Alexa Fluor 488, 1:500, anti-rabbit Alexa Fluor 568, Invitrogen Co. Carlsbad, CA, USA) and DAPI (DAKO, Carpinteria, CA, USA) for 30 min. The stained embryos were observed under a confocal microscope (ZEISS. Oberkochen, Germany).

### 2.5. Electron Microscopy

For transmission electron microscopy, zebrafish (*n* = 20) were fixed with 2.5% glutaraldehyde (Nacalai Tesque Inc. Kyoto, Japan) in 0.1 M phosphate buffer (pH 7.4); this was followed by post-fixation with 1% OsO_4_ in the same buffer. The fixed specimens were dehydrated using a graded series of ethanol and embedded in Epok812 (Oken Shoji. Tokyo, Japan). Ultrathin sections were prepared and stained with uranyl acetate and lead citrate. The specimens were examined using an HT7700 transmission electron microscope (Hitachi. Tokyo, Japan).

### 2.6. Cell Culture and Differentiation

C2C12 cells were purchased from KAC (Kyoto, Japan). Cells were cultured in Dulbecco’s Modified Eagle Medium (DMEM) (Sigma-Aldrich Co. St. Louis, USA) containing 10% fetal bovine serum (FBS) (Invitrogen Co. CA, USA), and incubated at 37 °C in a humidified chamber with 5% CO_2_. The cells were incubated with or without 2-fluorofucose (2FF) (Keyman chemical, USA) dissolved in DMSO. The final concentrations of 2FF were diluted with culture media at 0.5 and 1 mM. Culture medium containing an equal amount of DMSO (Sigma Aldrich, USA) was used as a control. When cells became confluent, the medium was changed to 2% horse serum (Invitrogen, USA)/DMEM. Cells were cultured for up to 5 days with the medium being changed every 2 days to induce differentiation.

### 2.7. Fusion Index and Differentiation Index

On day 5 of differentiation induction, cells were fixed in 4% PFA (Nacalai Tesque Inc. Kyoto, Japan) for 15 min at RT, incubated with 0.2% Triton X-100 (Sigma-Aldrich Co. St. Louis, USA), and blocked using Immunoblot (Nakarai, Japan). All samples were incubated with MY-32 (1:300, Sigma-Aldrich Co. St. Louis, USA) and DAPI (DAKO. CA, USA).

The fusion index was calculated as the ratio of the nuclei number in myotube (MyHC-positive) with two or more nuclei versus the total number of nuclei. The differentiation index was calculated as the ratio of the nuclei number in myotube (MyHC-positive)/Total number of nuclei.

### 2.8. Total Protein Extraction

Total proteins were extracted using RIPA lysis and extraction buffer (Thermo Fisher Scientific. Commonwealth of Massachusetts, USA) containing Halt Protease Inhibitor Cocktail (100X) (Thermo Fisher Scientific. Commonwealth of Massachusetts, USA).

### 2.9. Lectin Blotting Analysis

Whole zebrafish and C2C12 cell lysates (10 μg/lane) were subjected to 10% sodium dodecyl sulfate-polyacrylamide gel electrophoresis (SDS-PAGE), followed by transfer to PVDF membranes. The membranes were blocked with 5% BSA in TBST overnight at 4 °C and incubated with 0.5 μg/mL of biotinylated aleuria aurantia lectin (AAL) (Seikagaku Corp. Tokyo, Japan), which preferentially recognizes Fuc-α-1,6-GlcNAc structures, in TBST for 1 h at 25 °C. After washing with TBST three times, lectin-reactive proteins were detected using a Vectastain ABC kit (Vector Laboratories Inc. San Francisco, USA) and an ECL kit (Amersham Biosciences. Chicago, USA).

### 2.10. Quantitative Real-Time PCR (qPCR)

Total RNA was extracted from C2C12 cells and zebrafish using Trizol reagent (Invitrogen Co. CA, USA), and cDNA was synthesized using the ReverTra Ace kit (TOYOBO Co., Ltd. Osaka, Japan). Gene expression was assessed using SYBR Green Master Mix (Applied Biosystems. Commonwealth of Massachusetts, USA). Analyses were performed using QuantStudio 3 (Applied Biosystems. Commonwealth of Massachusetts, USA) with the following SYBR primers: *Myomaker*-F:5′-ATCGCTACCAAGAGGCGTT-3′, *Myomaker*-R:5′-CACAGCACAGACAAACCAGG-3′, *Myomerger*-F:5′-CAGGAGGGCAAGAAGTTCAG-3′, *Myomerger*-R:5′-ATGTCTTGGGAGCTCAGTCG-3′, *Ckm*-F:5′-ACCTCCACAGCACAGACAGA-3′, *Ckm*-R:5′-CAGCTTGAACTTGTTGTGGG-3′, *Myogenin*-F:5′-CTACAGGCCTTGCTCAGCTC-3′, *Myogenin*-R:5′-GTGGGAGTTGCATTCACTGG-3′, r18s-F:5′-TCAAGAACGAAAGTCGGAGG-3′, and *r18s*-R:5′-GGACATCTAAGGGCATCACA-3′, *dag1*-F:5′-ACGATGCCCAGCCTTTCATCTG-3′, *dag1*-R:5′-GGCAAGTAGTTCCACCCTCTGC-3′, *jam2a*-F:5′- GGAGAAAATGCTGGTGTGCGTG-3′, *jam2a*-R:5′-TTAGGATTGCGACTGCTGACGG-3′, *gapdh*-F:5′-GTGGAGTCTACTGGTGTCTTC-3′, *gapdh*-R:5′- GTGCAGGAGGCATTGCTTACA-3′.

### 2.11. Statistical Analyses

Data are presented as mean ± standard deviation (SD). GraphPad Prism ver. 7 (GraphPad Software. San Diego, CA, USA) was used for the statistical analyses. Statistical significance was set at *p* < 0.05.

## 3. Results

### 3.1. Zebrafish fut8a Morphant Morphology

**Figure 1:**
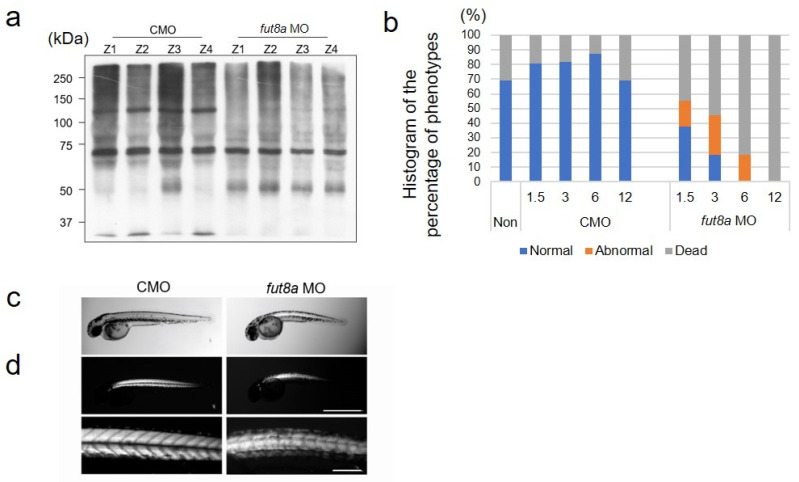
Zebrafish injected with *fut8a* MO showed abnormalities in skeletal muscle (**a**) Lectin blot, with AAL, of Zebrafish morphants injected with 3 ng of CMO or *fut8a* MO at 2 dpf. Total protein was visualized by Coomassie Blue staining and Ponceau staining (Supplemental Figure S1a,b): Histogram of the percentage of normal, abnormal, and dead fish in non-injected fish, CMO and *fut8a* MO injected fish at 2 dpf. Blue: normal, orange: abnormal, gray: dead. (**c,d**) Comparison of the CMO and *fut8a* MO injected zebrafish under bright field (**c**) and birefringence assay (**d**) at 2 dpf. d: Upper bar = 1 mm; lower bar = 200 μm. Non: non-injected zebrafish.

We used *fut8a* MO, whose effects have been reported previously [16]. Fut8a catalyzes the reaction that transfers fucose to the N-linked sugar chain, GlcNAc at the α-1,6 linkage. First, we confirmed whether *fut8a* MO effectively reduced *fut8a* expression, using AAL staining performed at 2 dpf (day post-fertilization) to detect α-1,6 linked fucose-added proteins. We observed that control morphants reacted strongly with a wide range of proteins ranging from 50–250 kDa, especially proteins of ~130 kDa. In contrast, *fut8a* morphants showed markedly reduced fucose-added proteins ranging from 100–250 kDa (Figure 1a). Next, to determine the effect of fut8a on zebrafish embryogenesis, we analyzed the mortality and morphology of control and *fut8a* morphants at 2 dpf. Control morphants showed mortality rates of 19.0% at 1.5 ng CMO, 18.2% at 3 ng, 12.5% at 6 ng, and 31% at 12 ng. Therefore, there was no correlation between the CMO dose and mortality (Figure 1b). In contrast, nearly half of the *fut8a* morphants died even at low concentrations of *fut8a* MO with 44.8% lethality at 1.5 ng. Furthermore, the mortality increased in a dose-dependent manner (3 ng: 54.5% and 6 ng: 81.4%). All fish injected with 12 ng *fut8a* MO died (Figure 1b). Subsequent experiments were performed on animals at 2 dpf; they were injected with 3 ng of CMO or *fut8a* MO. Zebrafish treated with *fut8a* MO showed obvious developmental defects, such as small body size and curvature of the spine (Figure 1c). Next, the muscle structure was analyzed using the birefringence assay for more detailed observation of myofiber structure and degeneration. *fut8a* morphants showed significantly reduced birefringence compared to control morphants, indicating an abnormal skeletal muscle structure (Figure 1d). In contrast, all control morphants showed normal morphology. 

### 3.2. fut8a Expression Is Required for the Formation of Myosepta

**Figure 2:**
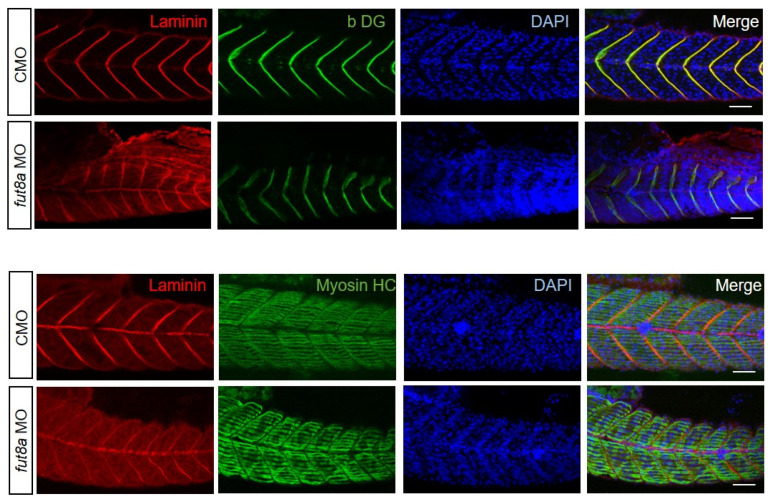
Reduced *fut8a* in zebrafish triggers skeletal abnormalities anti-laminin, anti-beta-dystroglycan (βDG), anti-myosin heavy chain (Myosin HC), and DAPI. Scale bar: 50 μm.

**Figure 3:**
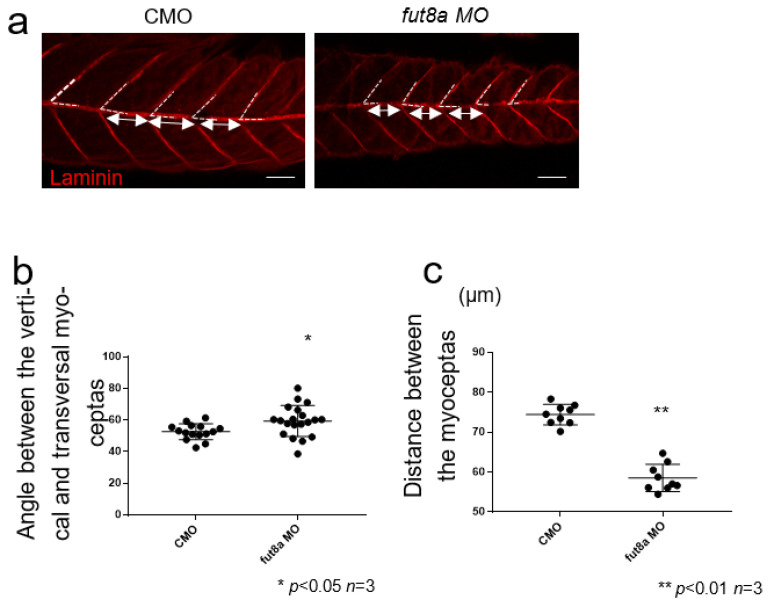
*fut8a* reduction causes structural abnormalities in myosepta (**a**) Left panel is CMO, right panel is *fut8a* MO. The white dotted line indicates the angle between the dorsal vertical and transverse myoseptas, and the double arrow indicates the distance of the transverse myoseptas, stained with anti-laminin. Scale bar: 50 μm. (**b**) Histogram of the angle between the vertical and transverse myoseptas. (**c**) Histogram of the variation in sarcomere length. Five to ten sarcomere lengths were calculated from five fish. CMO: Median 1.956, Max 2.032, Min 1.869. *fut8a* MO: Median 2.0325, Max 2.153, Min 1.772 (*t*-test. *p* < 0.01, *n* = 5).

We performed immunohistochemical staining using antibodies, including anti-laminin, anti-beta-dystroglycan, and anti-myosin heavy chain (MHC, slow muscle), to examine the expression pattern of muscle proteins. Zebrafish muscle tissue consists of blocks (myomeres) attached to connective tissue called myosepta, which correspond to tendons, and provide mechanical support for the muscle. *Fut8a* morphants showed abnormal myosepta formation compared to control morphants (Figure 2). Immunohistochemical staining of control morphants and *fut8a* morphants at 2 dpf was performed using an anti-laminin antibody to measure the angle of the dorsal vertical myosepta and transverse myosepta at five sites per zebrafish, and the mean angles were determined. The results showed a significantly lower angle of 52.59 ± 5.04° in control morphants, and 59.38 ± 9.74° in *fut8a* morphants (*p* < 0.05, *n* = 3) (Figure 3a,b). Similarly, the distance between transverse myosepta was measured at three locations, and the mean distance was found to be 74.40 ± 6.450 μm for control morphants, and 58.54 ± 11.69 μm for *fut8a* morphants (*p* < 0.01 *n* = 3). The distance between the transverse myosepta of *fut8a* morphants was significantly reduced (Figure 3a,c). These results suggested that fut8a expression is required for the formation of myosepta in zebrafish.

### 3.3. Microstructural Analysis Using Electron Microscopy

**Figure 4:**
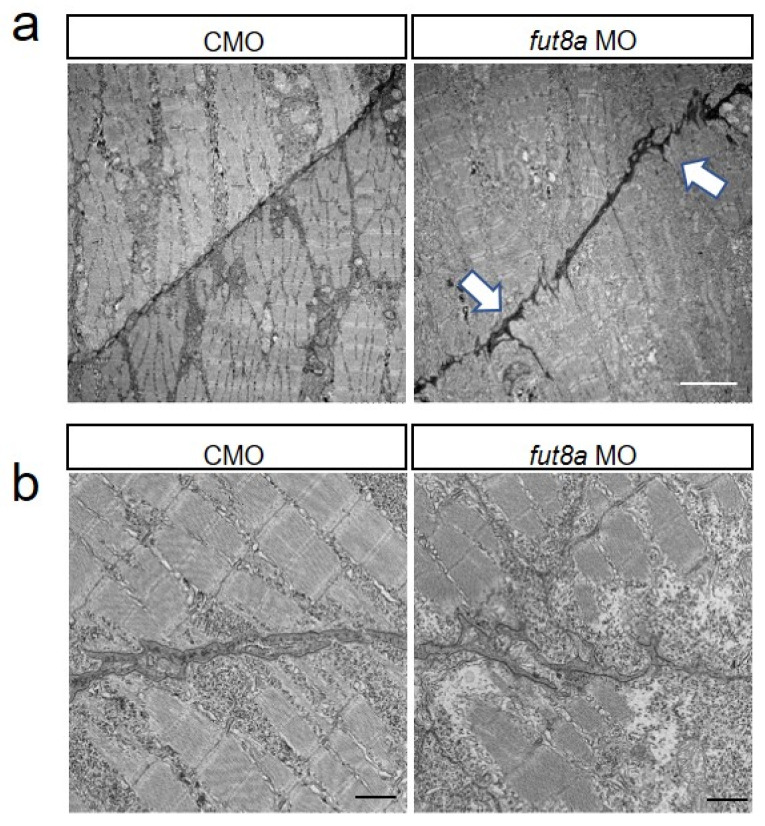
*fut8a* morphants have abnormal myosepta Representative electron micrographs of longitudinal section of morphants (2 dpf). (**a**) Electron micrograph of dorsal myosepta. White arrows indicate the tear sites in the myosepta. Scale bar: 5 μm. (**b**) Enlarged views of electron micrograph of dorsal myosepta. Scale bar: 1 μm. Maximum width, total length, and straightness factor of myosepta were quantified and indicated in Supplemental Figure S2.

The abnormal formation of myosepta, the attachment sites of myofibers, could be due to abnormalities in myofibers. Thus, we investigated the microstructure of the binding sites of the myosepta and myofibers using electron microscopy. We observed linear myosepta in control morphants, while the myosepta in *fut8a* morphants showed multiple areas of breakage (Figure 4a). The myosepta were bound to myofibers in controls, while *fut8a* morphants displayed disrupted myosepta binding sites (Figure 4b).

**Figure 5:**
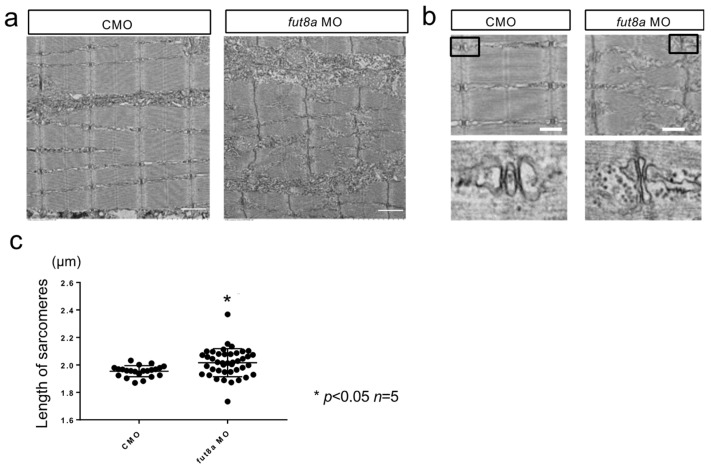
*fut8a* morphants show abnormalities in sarcomere formation (**a**) *fut8a* morphants displayed abnormal sarcomere structure with distorted Z-line and H-bands. Scale bar: 1 μm. (**b**) The triad of *fut8a* morphants was disorganized. Black boxes show the location of lower images. Scale bar: 1 μm. (**c**) Histogram of the variation in sarcomere length. CMO: Median 1.956, Max 2.032, Min 1.869. *fut8a* MO: Median 2.0325, Max 2.153, Min 1.772 (*t*-test. *p* < 0.01, *n* = 5).

The sarcomere structure of *fut8a* morphants was analyzed for changes in myofibers, because the binding sites between myosepta and myofibers were disrupted in these morphants. We observed normally aligned Z and H bands in control morphants but disrupted Z line and absent H band in *fut8a* morphants (Figure 5a). Additionally, *fut8a* morphants also showed abnormal T-tubule formation and sarcomere structure (Figure 5b). We measured sarcomere lengths of control and *fut8a* morphants to quantify variations in the sarcomere structure. The sarcomeres of the control morphants were almost 2 µm in length, whereas those of the *fut8a* morphants varied significantly, from 1.7 to 2.4 µm in length (Figure 5c, *p* < 0.01, *n* = 5). Thus, loss of fut8a leads to abnormalities in the microstructure of the myosepta and sarcomeres.

### 3.4. Fut8 Facilitates Muscle Differentiation

**Figure 6:**
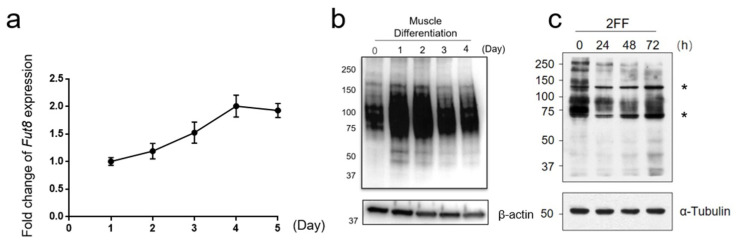
Altered expression and fucosylation of *Fut8* in C2C12 cells (**a**) *Fut8a* expression during C2C12 cell differentiation. Relative control normalized by r18 on day 1. (b) Lectin blot, with AAL and anti-β-actin, of C2C12 cells with induced muscle differentiation. (**c**) Lectin blot, with AAL and anti-α-tubulin, of C2C12 cells treated with 1 mM 2-Fluorofucose (2FF). * Nonspecific band.

We performed in vitro experiments using C2C12 cells—an immortalized mouse myoblast cell line—to confirm Fut8 function in myogenesis. We observed an increased *Fut8* expression with the progression of muscle differentiation, eventually increasing approximately 2-fold compared to pre-differentiation levels (Figure 6a, *n* = 3). Next, we investigated the degree of fucosylation of proteins modified by Fut8, using AAL staining. We observed rapidly increasing fucosylated protein levels upon muscle differentiation induction, starting on day 1 of induction, and remaining at high levels thereafter (Figure 6b). 

**Figure 7:**
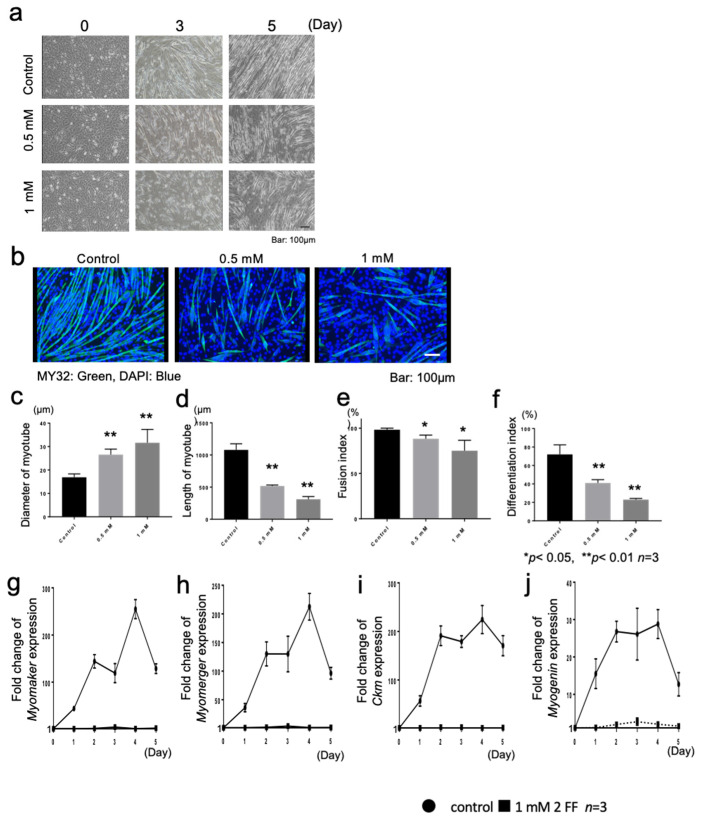
Inhibition of Fut8 function by 2FF inhibits myotube formation (**a**) Changes in muscle differentiation after the addition of 0, 0.5, and 1 mM 2FF. Scale bar: 100 μm (**b**) Immunostaining of C2C12 cells on day 5 of induced muscle differentiation using anti-MY32. Scale bar: 100 μm. (**c**) Diameter of myotube (one-way ANOVA, *p* < 0.01, *n* = 3). (**d**) Length of myotube (one-way ANOVA, *p* < 0.01, *n* = 3). (**e**) Histogram of fusion index (one-way ANOVA, *p* < 0.05). (**f**) Differentiation index (one-way ANOVA, *p* < 0.05). (**g**–**j**) Quantitative RT-PCR using the delta-delta Ct method for *Myomaker*, *Myomerger*, *Ckm*, *and Myogenin* expression during differentiation of C2C12 cells. Relative control normalized by r18 and Day 0 (*n* = 3).

We then attempted to elucidate the function of Fut8 during myogenesis using 2FF, an analog L-fucose that selectively inhibits core fucosylation by interfering with the normal synthesis of GDP-fucose [21,22]. The activity of 2FF has been previously confirmed in hepatoma cell lines [23] and in pancreatic cancer cell lines [24]. First, fucosylated proteins were investigated using AAL staining to determine whether the addition of 2FF reduced fucosylated proteins (Figure 6c). We next confirmed the effect of *Fut8* on muscle differentiation by adding 0.5 and 1 mM of 2FF during C2C12 cell differentiation. Induction of myogenesis with 2FF suppressed myotube formation on days 3 and 5 compared to the untreated group (Figure 7a). We then examined C2C12 cells on day 5 of induced differentiation with 2FF. We found that myotubes supplemented with 2FF became significantly thicker, in a dose-dependent manner (Figure 7c, one-way ANOVA *p* < 0.01, *n* = 3). In contrast, myotube cell lengths were significantly shorter (Figure 7d, one-way ANOVA *p* < 0.01, *n* = 3).

Fusion index, a measure of myotube formation, was examined. The fusion index of C2C12 cells without 2FF was almost 100%, whereas the fusion index of C2C12 cells with 1 mM 2FF was significantly reduced to 70% (Figure 7e, one-way ANOVA *p* < 0.05, *n* = 3). The differentiation index in the control (or 2FF-naïve) group was 75%, but it was significantly reduced by 2FF in a dose-dependent manner, to 40% at 0.5 mM and 20% at 1 mM (Figure 7f,d: one-way ANOVA *p* < 0.01, *n* = 3).

Finally, we investigated the altered expression of genes involved in myotube formation, by inhibiting Fut8 function using 2FF. The expression of *Myomaker* and *Myomerger*, which are muscle-specific membrane proteins expressed at the time of myoblast fusion, increased more than 100-fold in the 2FF-naïve group, compared to before muscle differentiation induction and only approximately 4-fold in the 2FF-exposed group (Figure 7g,h). Similarly, the expressions of genes such as *Myogenin* and *Ckm*, which are upregulated during myoblast differentiation into myotubes, were not upregulated in the 2FF-treated group (Figure 7i,j).

## 4. Discussion

Proteins are biosynthesized through transcription and translation, followed by post-translational modifications such as glycosylation, methylation, and lipid addition. Glycosylation, a type of post-translational modification, involves the addition of sugars to proteins or lipids. Most membrane proteins, such as receptors, are glycosylated in the Golgi apparatus [3]. Fut8, the enzyme we focused on in this study, is the only α-1,6-fucosyltransferase enzyme responsible for core-fucosylation [7]. The symptoms and molecular mechanisms of human diseases caused by Fut8 dysfunction have not yet been fully elucidated, and clinical symptoms such as hypotonia suggest that it is important to verify muscle abnormalities in addition to abnormalities in the nervous system. Although dysfunction of Fut8 has been reported in humans, mice, and zebrafish, there have been no reports analyzing muscle function. Therefore, we investigated the effect of fut8a on myogenesis in zebrafish injected with MO and C2C12 cells.

Unlike mammals, fucose-containing proteins are not abundant in zebrafish and are difficult to detect by tandem mass spectrometry [25,26]. However, 2-dpf zebrafish injected with *fut8a* MO showed a remarkably reduced fucose addition to proteins (Figure 1a). Therefore, we analyzed the skeletal muscles of 2-dpf zebrafish injected with fut8a MO.

Microstructural analysis of *fut8a* morphants using electron microscopy revealed abnormalities in myosepta and sarcomere structures. Zebrafish undergo muscle maturation after hatching [20], and myosepta and myofibrils are not fully attached at 2 dpf. We could not verify whether the myosepta or myofibrils were affected in *fut8a* morphants that died at 2 dpf. 

*Fut8a* morphants injected with low concentrations of *fut8a* MO showed dorsal deformity and stunted development, suggesting that fut8a is required for myogenesis in zebrafish.

Humans and mice with *Fut8* mutations often die at a young age [13,14]. However, the cause of death in *Fut8* mutant individuals has not yet been elucidated [10,13,14]. Since the *fut8a* morphants that died also failed to hatch, myogenesis may have been affected in these individuals; however, the cause is unclear. As previously reported, *Fut8* is expressed in various tissues. In particular, *Fut8* has a profound influence on the development and maturation of the brain and nervous system [27]. Neural and muscle maturation are closely intertwined, making it important to determine whether these abnormalities, caused by reduced Fut8, are myogenic or neurogenic.

The gene expression of *dystroglycan* (*dag1*), which is involved in stabilizing skeletal muscle structure [28,29], and the expression of *junctional adhesion molecule 2a* (*jam2*), which is involved in cell membrane fusion [30], were also significantly reduced, to <20%, compared with the CMO (Supplemental Figure S3). Dystroglycan binds to intracellular actin cables and participates in maintaining the structural integrity of myofibers by linking the extracellular matrix and actin cables as part of a dystroglycan complex [31].

Zebrafish is a useful model for analyzing development. Whole body MO knockdown was used in this study, which disabled the characterization of tissue specificity. To analyze muscle specific role of Fut8, a Fut8 inhibitor, 2FF, was used to investigate the effects of Fut8 inhibition in C2C12 cells in vitro [23].

Upregulation of *Myomaker* and *Myomerger* expression was lower in 2FF-treated C2C12 cells (Figure 7g,h) compared to untreated C2C12 cells during differentiation. Abnormalities in the actin cytoskeleton cause disruption of *Myomaker* and *Myomerger* expression [32]. Actin is an intracellular microfilament, and integrins and receptor tyrosine kinases lie upstream of extracellular signaling [33]. Both receptor tyrosine kinases and integrins are target proteins of Fut8 [8,34], suggesting that the effects we observed were caused by protein destabilization and inhibition of intercellular signaling, due to the lack of core fucosylation on the target proteins of Fut8. These results are consistent with the phenotype observed in *fut8a* morphants and with the in vitro experiments performed with C2C12 cells [8,34], suggesting that the effects we observed were caused by protein destabilization and inhibition of intercellular signaling that resulted from the lack of core fucosylation on the target proteins of Fut8. Generation and analysis of skeletal muscle-specific Fut8-deficient mice is required to identify target proteins of Fut8 and accurately confirm the function of Fut8 in skeletal muscle. 

## Supplementary Materials

The following supporting information can be downloaded at: https://www.mdpi.com/article/10.3390/cells12010144/s1, Figure S1: Total protein of Zebrafish morphants. Figure S2: Myosepta of *fut8a* morphants. Figure S3: Quantitative RT-PCR for *dag1* and *jam2a* expression.
