# Supplementary material for: α-1,6-Fucosyltransferase Is Essential for Myogenesis in Zebrafish
Source: Cells. 2022 Dec 29;12(1):144. doi: 10.3390/cells12010144 (PMC9818595; doi:10.3390/cells12010144)
Supplement: Supplementary file 1 [file cells-12-00144-s001.zip › cells-1995386-supplementary.pdf]

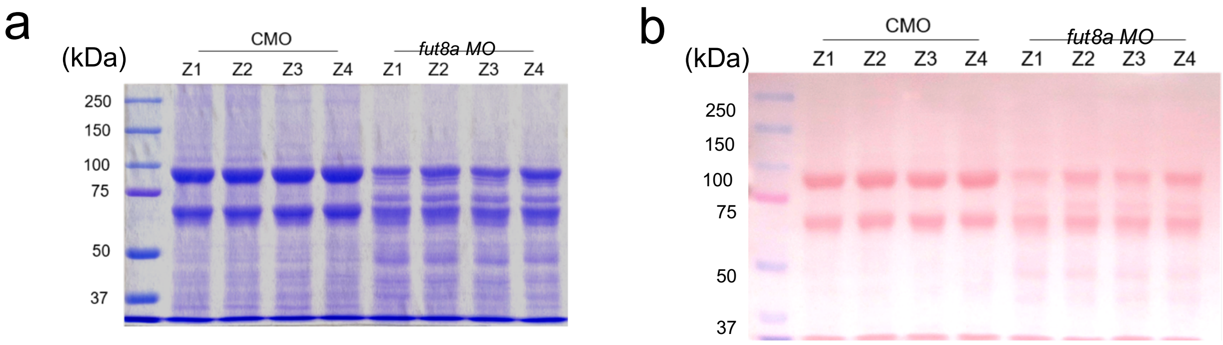

Supplemental Figure S1 Total protein of Zebrafish morphants.

A and B: Zebrafish morphants injected 3 ng CMO and *fut8* MO at 2 dpf total protein were visualized by Coomassie Blue staining and Ponceau staining.

Supplemental Materials and Methods

Coomassie Brilliant Blue Staining

Shake and wash the gel with deuterium-depleted water (DDW)for 5 min x 3 times at room temperature. Add Quick-CBB Plus (Wako, Japan) and shake for 30 min until protein bands are visible. Discard the stain and wash with DDW for 60 min.

Ponceau S Staining

Rinse membrane in DDW briefly. Incubate the membrane in Ponceau S Staining Solution (CST, USA) for 10 min at room temperature. Wash the membrane in DDW and TBS-T 5 min x 3 times.

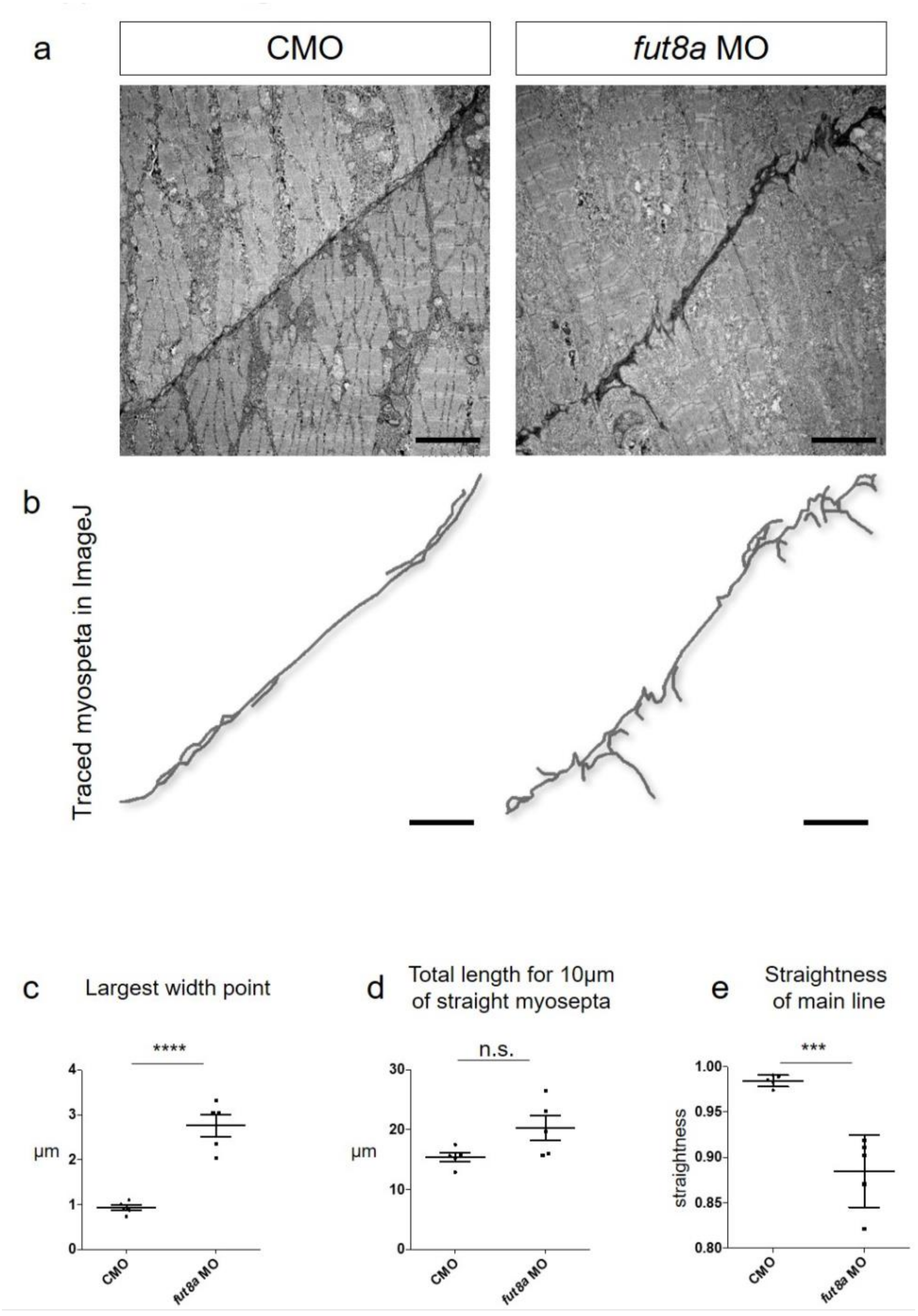

Supplemental Figure S2. *fut8a* morphants myosepta is ruptured. a, Electron micrograph of dorsal myosepta. b, reconstructed skeleton of mysepta in ImageJ software. c, maximum width of myosepta observed in electron micrographs. d, total length of myosepta within a 10μm straight line. e, straightness factor of myosepta, 1 being the value for a straight line. Unpaired t test. Scale bar: 5μm. *p*: <0.0001 in c; 0.0597 in d; 0.0006 in e.

a

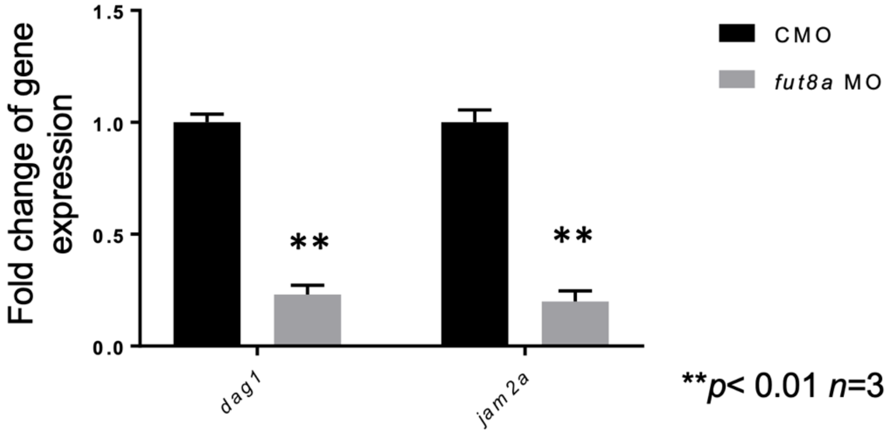

Supplemental Figure S3. Quantitative RT-PCR using delta delta Ct method for *dag1* and *jam2a* expression of CMO and *fut8a* morphants. Relative control normalized by *gapdh* (t-test, ** $p < 0.01$, $n=3$).
